# Methods for evaluation of helium/oxygen delivery through non-rebreather facemasks

**DOI:** 10.1186/2045-9912-2-31

**Published:** 2012-12-17

**Authors:** Andrew R Martin, Ira M Katz, Yonatan Lipsitz, Karine Terzibachi, Georges Caillibotte, Joëlle Texereau

**Affiliations:** 1 Delaware Research and Technology Center, American Air Liquide, Newark, DE, 19702, USA; 2 Medical Gases Group, Air Liquide Santé International, Les Loges-en-Josas, 78354, France; 3 Department of Mechanical Engineering, Lafayette College, Easton, PA, 18042, USA

**Keywords:** Facemask, Patient Interface, Helium, Oxygen, Heliox, Bench testing, Test lung, Face model, Head model, Manikin

## Abstract

**Background:**

Inhalation of low-density helium/oxygen mixtures has been used both to lower the airway resistance and work of breathing of patients with obstructive lung disease and to transport pharmaceutical aerosols to obstructed lung regions. However, recent clinical investigations have highlighted the potential for entrainment of room air to dilute helium/oxygen mixtures delivered through non-rebreather facemasks, thereby increasing the density of the inhaled gas mixture and limiting intended therapeutic effects. This article describes the development of benchtop methods using face models for evaluating delivery of helium/oxygen mixtures through facemasks.

**Methods:**

Four face models were used: a flat plate, a glass head manikin, and two face manikins normally used in life support training. A mechanical test lung and ventilator were employed to simulate spontaneous breathing during delivery of 78/22 %vol helium/oxygen through non-rebreather facemasks. Based on comparison of inhaled helium concentrations with available clinical data, one face model was selected for measurements made during delivery of 78/22 or 65/35 %vol helium/oxygen through three different masks as tidal volume varied between 500 and 750 ml, respiratory rate between 14 and 30 breaths/min, the inspiratory/expiratory ratio between 1/2 and 1/1, and the supply gas flow rate between 4 and 15 l/min. Inhaled helium concentrations were measured both with a thermal conductivity analyzer and using a novel flow resistance method.

**Results:**

Face models borrowed from life support training provided reasonably good agreement with available clinical data. After normalizing for the concentration of helium in the supply gas, no difference was noted in the extent of room air entrainment when delivering 78/22 versus 65/35 %vol helium/oxygen. For a given mask fitted to the face in a reproducible manner, delivered helium concentrations were primarily determined by the ratio of supply gas flow rate to simulated patient minute ventilation, with the inspiratory/expiratory ratio playing a secondary role. However, the functional dependence of helium concentration on these two ratios depended on the mask design.

**Conclusions:**

Large differences in mask performance were identified. With continued refinement, the availability of reliable benchtop methods is expected to assist in the development and selection of patient interfaces for delivery of helium/oxygen and other medical gases.

## Background

Inhalation of helium/oxygen (He/O_2_) mixtures has been explored as a means to lower the airway resistance and work of breathing of patients suffering from obstructive lung disease [1-6], and as a carrier gas to transport pharmaceutical aerosols to obstructed lung regions [7-12]. These effects stem from the physical properties of He/O_2_ mixtures, in particular their low density compared to air [13-16]. As supplied, the He concentration in therapeutic He/O_2_ mixtures typically ranges from 60% to 80%, so as to balance between the low density afforded by high He concentration and the patient’s supplemental O_2_ requirements. During noninvasive ventilation (NIV) combining He/O_2_ with pressure support [1,2], the tight fit of the NIV mask and the elevated pressure in the mask dead space combine to prevent entrainment of ambient room air and ensure that the targeted He concentration is inhaled by the patient. However, during unassisted spontaneous breathing, recent clinical investigations have highlighted the potential for room air entrainment to dilute He/O_2_ mixtures delivered through standard non-rebreather facemasks [17,18], thereby potentially limiting efficacy of He/O_2_ therapy and certainly confounding efforts to determine optimal He/O_2_ mixture concentrations when using non-rebreather masks.

The development of He/O_2_ delivery strategies aimed at reducing room air entrainment (preferably while conserving gas consumption) will potentially entail design of purpose-made facemasks or other patient interfaces, improved means of matching supplied gas flows to patient demand, and/or use of semi-closed breathing circuits. Moreover, novel patient interfaces and gas supply means will be required for other medical gases, for example as new applications for nitric oxide, or for sub-anesthetic delivery of nitrous oxide and xenon, reach clinical evaluation. In the early-stages of product development, the availability of bench apparatus for collection of test data is advantageous, provided those test data can be trusted to be representative of use in the clinical setting. Evaluating facemask designs on the bench is particularly challenging given patient variation in facial features and size, and tolerance of fit tightness. While face models have been examined for testing aerosol drug delivery through facemasks [19,20], little information is available on the suitability of face models for testing medical gas delivery interfaces.

The aim of the present study was to compare several approaches to evaluating He/O_2_ delivery through non-rebreather facemasks on the bench. Four different face models were initially studied, ranging from a flat plate to more anatomically realistic manikins borrowed from life support training. Data obtained on the bench were compared with those obtained by Roche-Campo et al. [17] in a set of six healthy volunteers. Subsequently, a single face model was selected for a wider investigation of three different mask designs over a range of supply gas flow rates and simulated patient breathing patterns.

## Methods

### Facemasks

**Figure 1:**
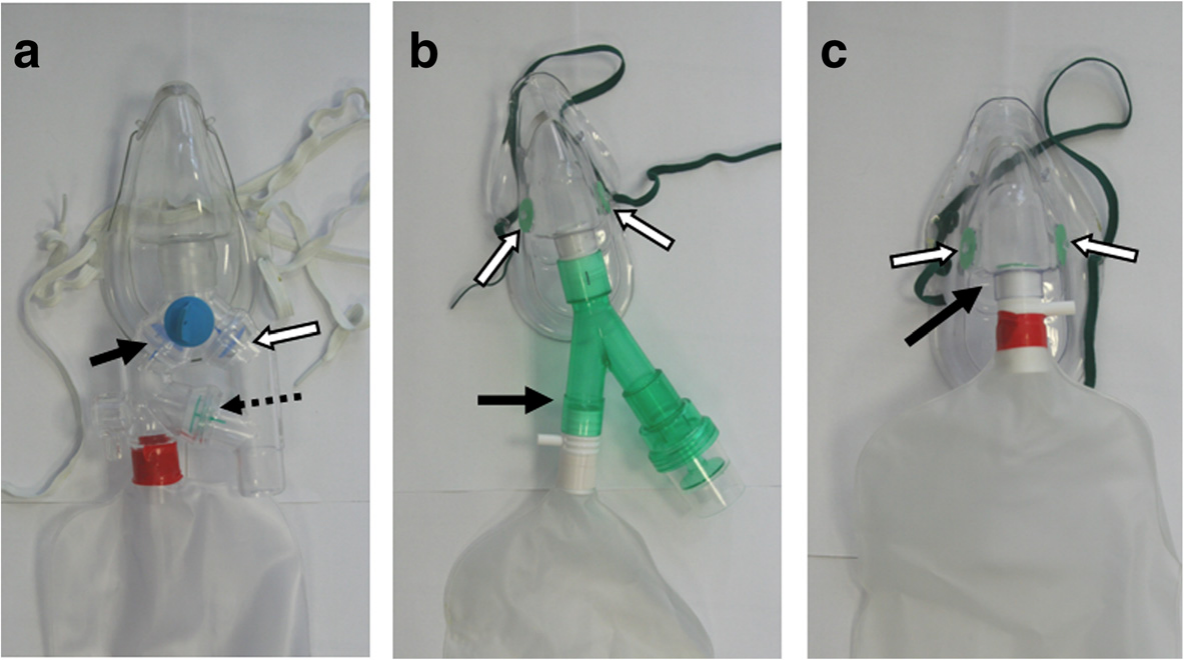
**Non-rebreather facemasks evaluated in the present study: a) The Hi-Ox mask, b) the Heliox21 mask, and c) the standard mask. Black arrows indicate the positions of inhalation valves, while white arrows indicate the position of exhalation valves.** The dotted arrow in **a**) indicates the position of the anti-suffocation valve.

Three non-rebreather facemasks were studied, as pictured in Figure 1. These included a Pulmanex Hi-Ox mask (Viasys Healthcare, Netherlands), a Heliox21 mask (Intersurgical, UK), and a standard, three-valve reservoir mask (Intersurgical, UK). While the standard and Hi-Ox masks were developed to deliver high oxygen concentrations, they have also been used for He/O_2_ administration.

### Bench apparatus

**Figure 2:**
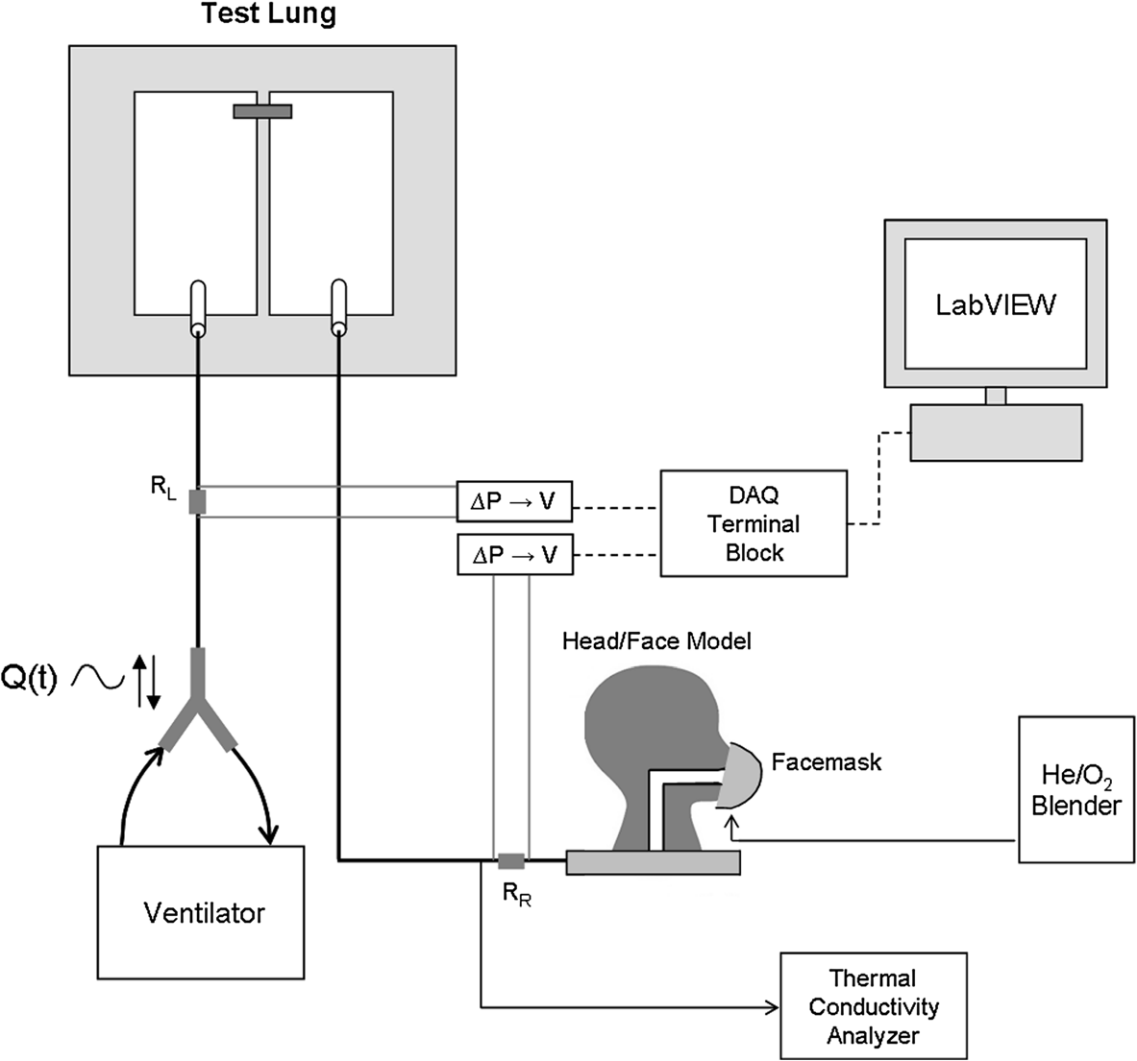
**Schematic of the experimental apparatus used to test helium/oxygen (He/O**_**2**_**) delivery through facemasks.** R_R_ and R_L_ represent parabolic resistors (R_p_5) placed in the flow paths supplying the right and left compartments of the test lung. ΔP → V represents transducers used to convert the differential pressure measured across either resistor to a voltage signal sent to the data acquisition (DAQ) system.

The experimental apparatus is shown schematically in Figure 2. Patient breathing was simulated using a dual chamber adult test lung (Michigan Instruments, USA) with the two chambers connected via a lifting bar. One chamber, the driving chamber, was connected to a ventilator (Neftis ICU; Taema, France) operated in volume control mode to impose breathing patterns, which varied in tidal volume between 500 and 750 ml, in respiratory rate between 14 and 30 breaths/min, and in the ratio of inspiratory time to expiratory time (t_i_/t_e_) between 1/2 and 1/1. All experiments were made with a constant (approximately square waveform) inspiratory flow. The second chamber, the breathing chamber, was connected to the various face models. Facemasks were fit to the face models, and He/O_2_ (Air Liquide, France) was supplied to the facemasks at either 78/22 or 65/35 %vol through a gas blender (Sentry He/O_2_ Blender; Cardinal Health, USA) at flow rates ranging from 4 to 15 l/min.

### Face models

Four different face models were evaluated. A s*ealed* model was constructed simply by fixing the facemasks to a flat face plate using adhesive putty to create a gas-tight seal between the mask cuff and the plate. A 22 mm hole was cut in the face plate to fit an inlet for the throat described below. Again, this inlet was sealed to the face plate using adhesive putty. An *open* model employed a hollow glass manikin head with a 40 mm diameter hole cut out at the position of the mouth. For both the open and sealed models, a ‘throat’ was constructed from standard 15 mm medical tubing, a 90° elbow connector, and an additional 15 mm ID/22 mm OD adapter used to extend the throat to create an inlet at the position of the mouth. The dead volume between the throat inlet and the position from which gas was sampled for analysis was 100 ml. For the open model, Parafilm (Fisher Scientific, France) was used to close-off the mouth opening of the glass manikin around the throat inlet.

**Figure 3:**
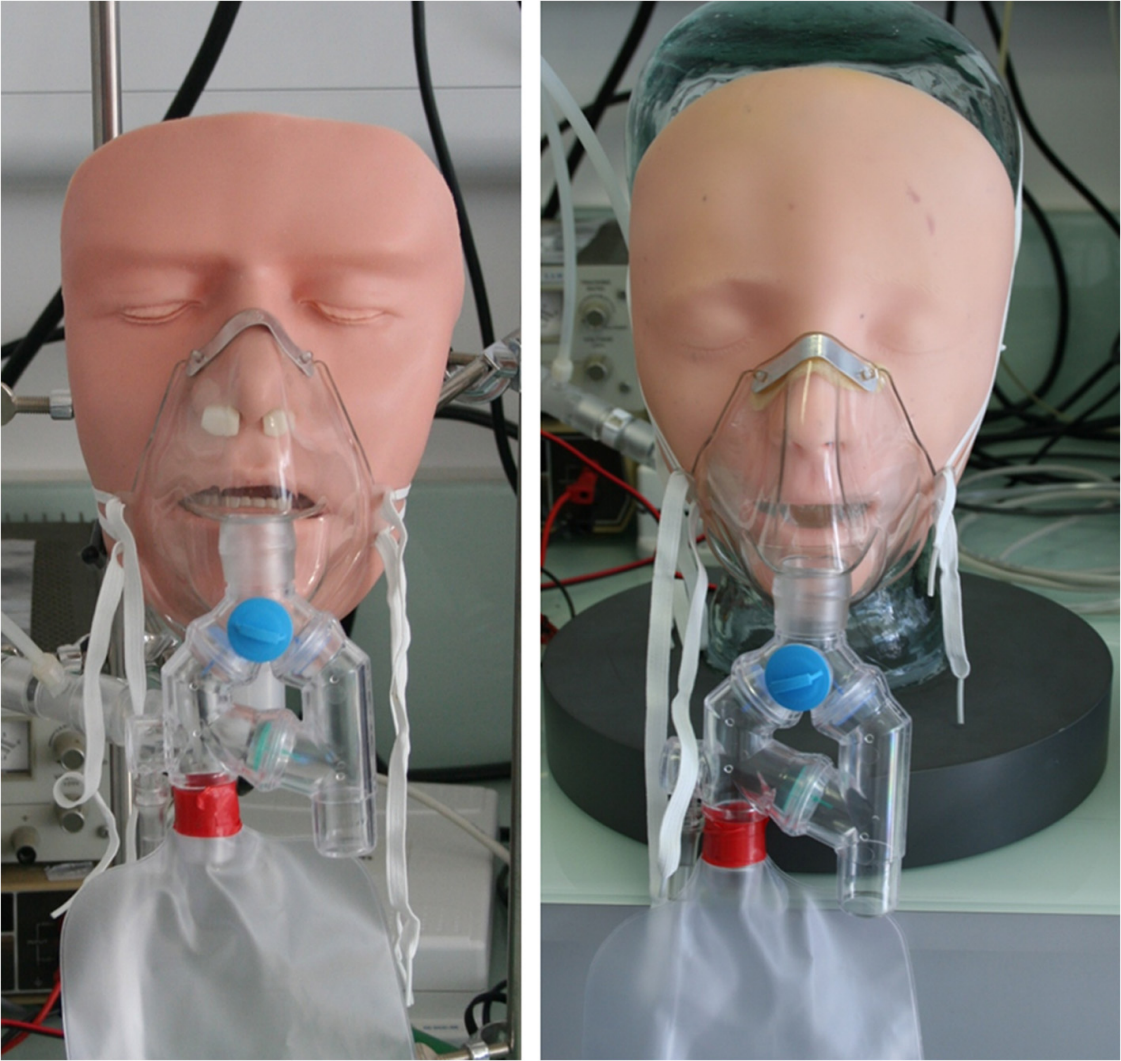
The Simulaids (left) and Laerdal (right) face models are shown fitted with the Hi-Ox mask.

Two face models used in life support training were also studied. For the *Laerdal* model, an adult manikin face (310210; Laerdal, Norway) was fitted over top of the glass manikin head as shown in Figure 3. The throat was kept in place, and the mouth opening of the manikin face was closed around the throat inlet, again using Parafilm, so as to simulate mouth breathing only. The *Simulaids* model employed a manikin face with upper airway (BLS Airway Trainer; Simulaids, UK), also shown in Figure 3. The nostrils of the model were plugged, and the outlet of the larynx was connected directly to tubing supplying the breathing chamber of the test lung, with the throat described above removed. The model had a second outlet, representing the esophagus, which was also plugged for all experiments. The total dead volume of the model was 230 ml.

### Helium concentration measurement

Two methods were used to measure delivered helium concentrations. A thermal conductivity analyzer (KG6050; Hitech Instruments, UK), calibrated to measure the helium concentration in binary helium-oxygen mixtures, drew a continuous sample of 200–300 ml/s from tubing supplying the breathing chamber of the test lung. As specified by the manufacturer, the T90 response time of this analyzer is 20 seconds; accordingly, for each experiment, the measured helium concentration was allowed to reach steady state over several breaths before being recorded.

In addition, a flow resistance technique was used to obtain intra-breath measures of the helium concentration. Parabolic resistors (Rp5, PneuFlow; Michigan Instruments, USA) were inserted into the flow paths supplying the two breathing chambers of the test lung. The differential pressure across each of these resistors was monitored and recorded using pressure transducers (PX277-05D5V; Omega Engineering Inc, USA) and LabVIEW data acquisition software (National Instruments, USA). The pressure drop across these resistors depends on both the gas flow rate and density [13]; accordingly, on the driving side of the test lung, where the gas density was known to be that of air, the resistor served as a differential pressure flow sensor. This flow measurement, along with the pressure drop measured across the resistor positioned on the breathing side, allowed the density (and in turn the helium concentration) of the gas on the breathing side to be determined, based on calibration data previously obtained by supplying gas to the resistor at known flow rates and helium concentrations. For all experiments, both the thermal conductivity analyzer and the flow resistor method were used to measure delivered helium concentrations.

When using the Laerdal and Simulaids face models, it was found in preliminary experiments that small adjustments made when fitting masks to the faces produced large changes in measured helium concentrations. Furthermore, the quality of the fit was difficult to assess visually. As a consequence, masks were fit to these face models while supplying He/O_2_ at 15 l/min and adjusting the fit to produce the highest possible helium concentration, as monitored using the thermal conductivity analyzer. The mask was then left in place and not adjusted while a series of measurements were performed at varying supply flow rate or breathing pattern.

## Results and discussion

### Comparison to clinical data

**Figure 4:**
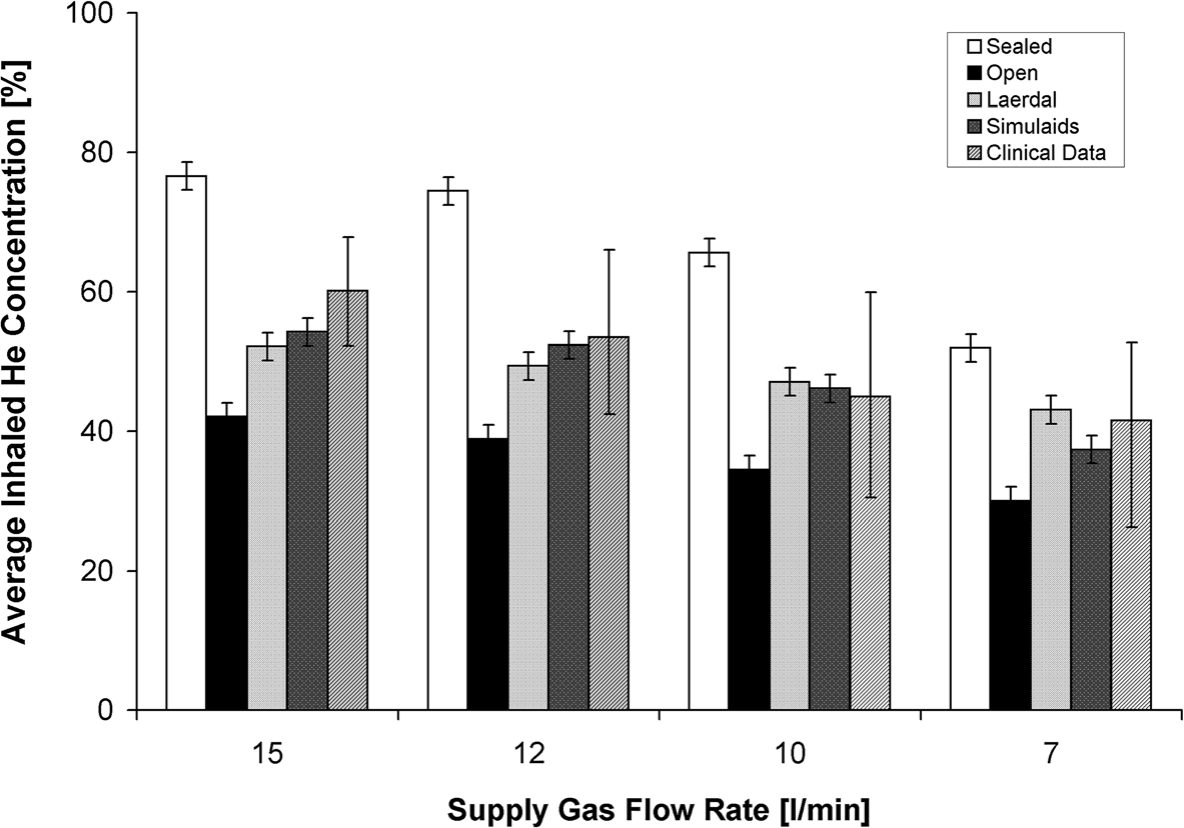
**Average inhaled helium concentrations for the Hi-Ox mask are displayed for a range of supply flow rates of helium/oxygen (He/O**_**2**_**) 78/22.** For the clinical data [17], error bars represent the limits of the range of values measured for six healthy, adult subjects. For all other columns, data was obtained from bench measurements made using the thermal conductivity analyzer. Error bars represent the ±2% accuracy of the analyzer.

Experiments were first performed with all four face models using the Hi-Ox mask supplied with 78/22 He/O_2_. These were made at a tidal volume of 500 ml and respiratory rate of 30 breaths/min to match the average breathing parameters recorded during the *in vivo* measurements performed on healthy adults using the same mask at resting conditions by Roche-Campo et al. [17]. The ratio t_i_/t_e_ was fixed at 1/2. Figure 4 compares the average inhaled helium concentrations measured with the thermal conductivity analyzer for the four face models with the mean and range of values measured *in vivo*. Concerning the face models, the sealed and open models represent upper and lower extremes in delivered helium concentrations, with values obtained for the Laerdal and Simulaids models falling in between. Little difference was observed between the average helium concentrations measured for the Laerdal and Simulaids face models, and concentrations measured using either of these models were in reasonable agreement with mean values measured *in vivo* in healthy adults. The series of experiments was repeated for the Heliox21 and standard facemasks using only the Laerdal and Simulaids models, and results are presented in Table 1. Again, agreement with mean values measured *in vivo* was good, though when testing the Heliox21 mask on the Simulaids model, helium concentrations delivered on the bench tended to lie just below the lower range of the *in vivo* dataset for the same mask.

**Table 1:** **Average inhaled helium concentrations measured on the bench and in healthy adult subjects**[17]

**Average inhaled He concentration**				
**Mask**	**Supply flow [l/min]**	**Laerdal [±2%]**	**Simulaids [±2%]**	**Clinical data [mean (range)%]**
Hi-Ox	15	52.2	54.3	60.1 (52.3 - 67.8)
	12	49.4	52.4	53.5 (42.5 - 66.0)
	10	47.1	46.2	45.0 (30.5 - 59.9)
	7	43.1	37.4	41.5 (26.3 - 52.8)
Heliox21	15	37.9	32.9	54.9 (35.4 - 69.9)
	12	34.1	28.9	46.1 (30.7 - 57.7)
	10	31.7	25.3	38.3 (31.1 - 47.0)
	7	27.2	20.6	28.2 (18.8 - 35.3)
Standard	15	36.5	42.1	50.9 (41.4 - 63.1)
	12	32.7	36.3	42.2 (30.5 - 54.6)
	10	29.6	31.6	36.7 (26.3 - 43.3)
	7	24.9	26.1	26.7 (20.9 - 32.3)

### Helium concentration measurement

**Figure 5:**
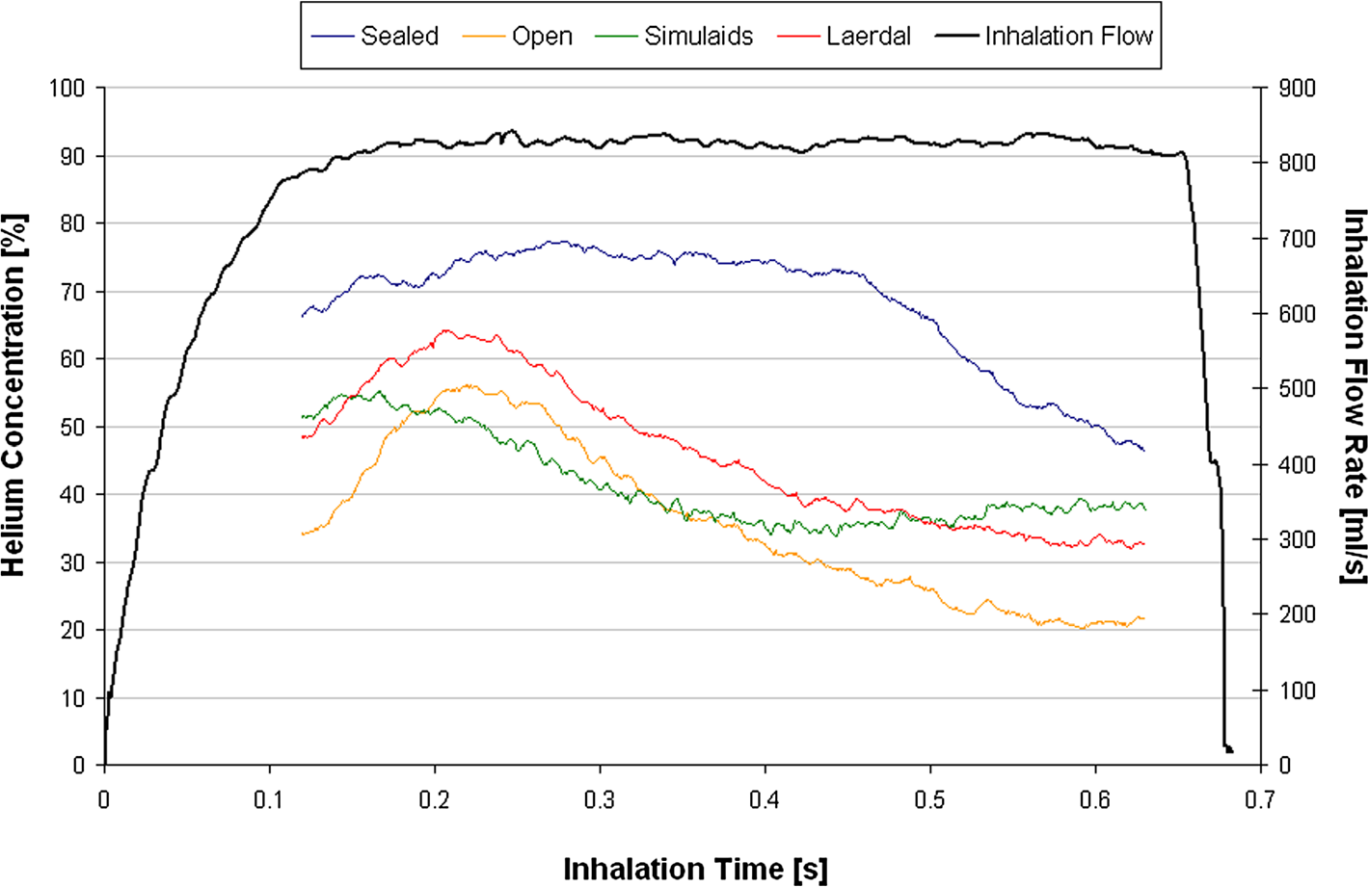
**An example of data obtained using the flow resistance method for determining inhaled helium concentrations, taken for the Hi-Ox mask supplied with 10 l/min of 78/22% helium/oxygen (He/O**_**2**_**), while ventilating at 30 breaths/min with a tidal volume of 500 ml and a ratio t**_**i**_**/t**_**e **_**of 1/2.** Each curve is an average of data obtained over seven consecutive breaths. Also shown is a representative trace showing the inspiratory flow pattern.

The response time of the thermal conductivity analyzer was sufficiently slow that measured values represent time-averaged helium concentrations over several breaths. In contrast, using the flow resistor method it was possible to resolve variation in inhaled helium concentration within a single breath. Example data is shown in Figure 5, which was taken for the Hi-Ox mask supplied with 10 l/min of 78/22% He/O_2_ and fit to each of the four face models. All data shown in Figure 5 were obtained at a tidal volume of 500 ml, a respiratory rate of 30 breaths/min, and a ratio t_i_/t_e_ of 1/2. Also shown in Figure 5 is a representative trace showing the inspiratory flow pattern. Note that helium concentration data are not reported in Figure 5 at the start and end of the breath. This is a limitation of the measurement approach; during periods of rapid flow rate increase or decrease, instantaneous flow rates in the driving and breathing sides of the test lung are not equal, with the breathing chamber generally lagging the driving chamber. As a result, the flow rate derived from the pressure drop measured across the resistor on the driving side is different from the flow rate on the breathing side, and will lead to errors if used in the derivation of the helium concentration as described above.

Nevertheless, several useful pieces of information are conveyed in Figure 5. First, even for the sealed model, the delivered helium concentration falls off towards the end of the breath. This should not be surprising considering the ratio of gas supply flow relative to the simulated patient demand. At a tidal volume 500 ml and respiratory rate of 30 breaths/min, the inhaled minute volume is 15 l/min. Accordingly, supplying 10 l/min to a non-rebreather mask requires that an additional 5 l/min of gas be entrained from the room every minute. With the mask tightly sealed to the faceplate, this can be considered a *controlled* room air dilution, in that dilution occurs through an anti-suffocation valve, positioned on the mask’s supply manifold, and towards the end of the breath, once the reservoir bag is emptied. For the other three face models studied, where leaks occurred between the mask cuff and the face, there was an additional *uncontrolled* dilution, for which the location and timing during the breath are not obvious. In these cases, the inhaled helium concentration never reached the 78% contained in the supply flow.

Still with reference to Figure 5, all four curves initially rise near the beginning of the breath before later falling. We hypothesize that the former effect occurred due to inhalation of gas from the combined dead volumes of the models and masks. The helium concentration in the dead volume would be that of gas expired from the breathing chamber, and as such would be lower than that of the source gas. Therefore, it is likely that inhaled helium concentrations initially rose as fresh gas from the source flow and reservoir bag washed into the dead volume, before peaking and then falling off as the reservoir emptied. Such an effect could also explain the different pattern seen for the Simulaids model as compared to the Laerdal and open models, with the flatter curve for the Simulaids model linked to its larger dead volume.

**Figure 6:**
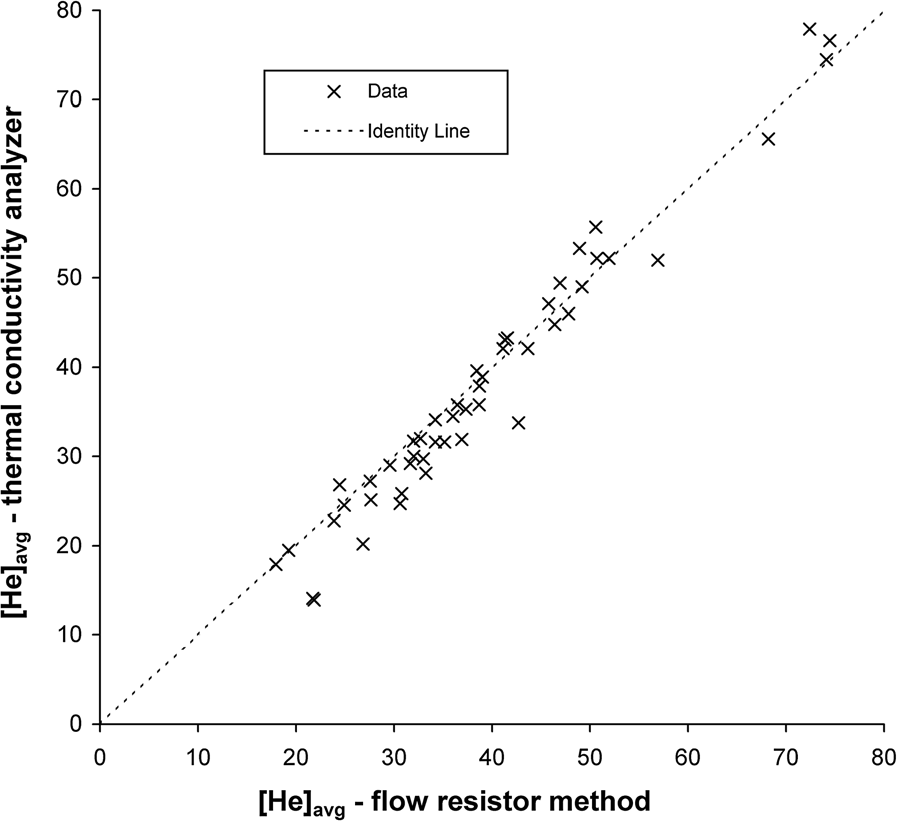
**The time-averaged inhaled helium concentration, [He]**_**avg**_**, measured using the flow resistance method is plotted against that measured by the thermal conductivity analyzer.**

Figure 6 compares helium concentrations measured using the flow resistor method and averaged over an inhalation with those measured by the thermal conductivity analyzer. As seen the two methods were in close agreement, which can be viewed as a validation of the flow resistor method, but also as evidence that the steady-state reading on the thermal conductivity analyzer did indeed represent a time-averaged helium concentration. Given its ease of use and commercial availability, the thermal conductivity analyzer seems a reasonable candidate for implementation of similar testing procedures across laboratories.

### Parameter study

**Figure 7:**
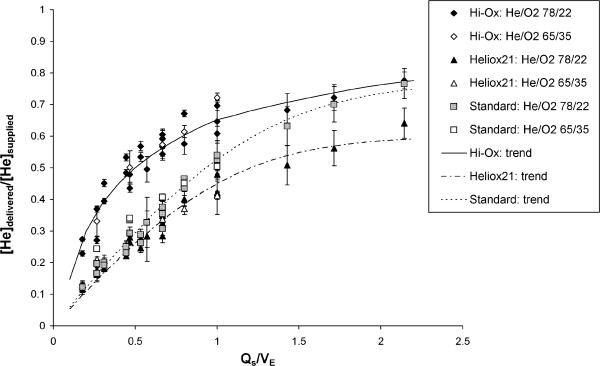
**The normalized delivered helium concentration, [He]**_**delivered**_**/[He]**_**supplied**_**, is plotted against the ratio between the supply gas flow rate and minute ventilation, Q**_**s**_**/V**_**E**_**, for each of the three masks tested, supplied with 78/22 or 65/35 %vol helium/oxygen (He/O**_**2**_**).** For comparison, trend lines are also shown for each mask. Error bars represent standard deviations around mean values (n = 3).

In an effort to further investigate the performance of the three masks, additional experiments were conducted over the full parameter range described in the Methods section, using only the Simulaids model. This model was chosen primarily because it was used as supplied by the manufacturer without modification, and as such would lend itself most easily to use in other laboratories. Figure 7 displays data from the full range of the experiments. Delivered helium concentrations are plotted against the ratio of supply flow rate to minute volume (Q_S_/V_E_). As experiments were conducted with both 78/22 and 65/35% He/O_2_, delivered helium concentrations were normalized by the supplied concentration. With this normalization, data obtained using either mixture appear to follow the same trend. That is to say, the relatively small differences in gas properties between 78/22 and 65/35% He/O_2_[16] did not influence room air entrainment, such that results obtained when testing with one He/O_2_ mixture can be readily extrapolated to predict inhaled concentrations that would be delivered when supplying a different He/O_2_ mixture, at least so long as the helium concentration in those mixtures ranges between 65 and 78%.

Again with reference to Figure 7, two regions of the curves can be defined. First, when the ratio Q_s_/V_E_ is less than one (that is when minute ventilation exceeds the supply flow rate) the masks can be considered to be undersupplied. In this case, the Hi-Ox mask outperformed both the standard mask and the Heliox21 mask. This is likely a result of the superior function of the expiratory valve on the Hi-Ox mask. As has been noted by previous investigators [21], expiratory flap-valves placed on standard masks permit room air entrainment during inhalation. By replacing these with a valve positioned in the gas supply manifold, which remains closed during inhalation, the design of the Hi-Ox mask promotes emptying of the reservoir prior to a controlled room air entrainment [21]. This sequential function was clearly observable when conducting the experiments, whereas for the other two masks the reservoir bags did not completely empty, even when the masks were undersupplied.

When the ratio Q_s_/V_E_ exceeded one, such that the masks can be said to be oversupplied, inhaled helium concentrations delivered through the standard mask approached those delivered through the Hi-Ox mask, while concentrations delivered through the Heliox21 mask remained lower. We hypothesize that as the gas supply becomes increasingly high relative to patient demand, the conserving function provided by the reservoir becomes less critical, and that instead it is the relative resistance of the supply flow path compared to that of uncontrolled leak paths that determines room air entrainment. On the Heliox21 mask the inspiratory valve is positioned further from the patient compared to the standard mask, and the resulting small increase in resistance between the fresh gas supply and the patient may promote entrainment of air through leak paths.

**Figure 8:**
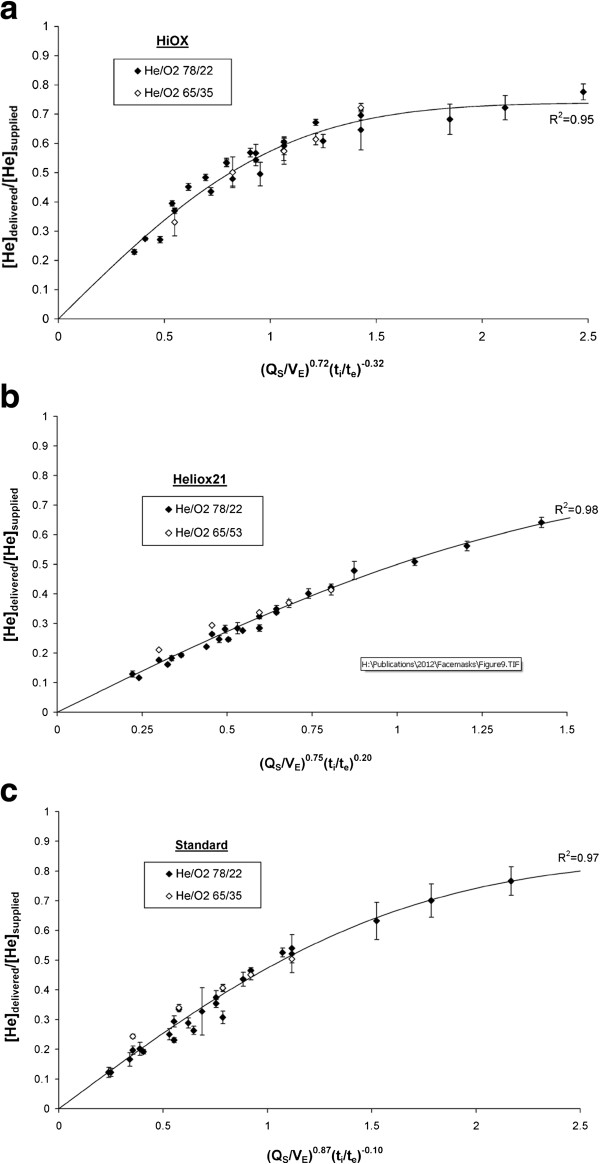
**The normalized delivered helium concentration, [He]**_**delivered**_**/[He]**_**supplied**_**, is plotted against a composite parameter that includes the ratio between the supply gas flow rate and minute ventilation, Q**_**s**_**/V**_**E**_**, and the ratio between inspiratory and expiratory times, t**_**i**_**/t**_**e**_**, for a) the Hi-Ox mask, b) the Heliox21 mask, and c) the standard mask.** Error bars represent standard deviations around mean values (n = 3). For each mask, the exponents in the composite parameter were determined through a least-squares fit of Equation (1) to the experimental data. Best-fit curves, and associated R^2^ values, are shown for each mask.

Differences in mask performance are further investigated in Figure 8. Data obtained for each mask were fit with functions of the form:

(1) $$\frac{{\left[\mathrm{He}\right]}_{\;\mathrm{delivered}}}{{\left[\mathrm{He}\right]}_{\;\mathrm{supplied}}}=A*\mathrm{erf}\left[B*{\left(\frac{Q_{s}}{V_{E}}\right)}^{C}{\left(\frac{t_{i}}{t_{e}}\right)}^{D}\right]$$

where the constants *A*, *B*, *C*, and *D* were determined for each mask by least-squares fitting, and are provided in Table 2. The error function (*erf*) is defined in the Appendix, and was selected here based solely on goodness of fit to the data in comparison with several other functions of similar shape.

**Table 2:** **Best-fit coefficients used in equation1for each facemask**

**Coefficient**				
**Mask**	**A**	**B**	**C**	**D**
Hi-Ox	0.7391	0.8601	0.7233	−0.3210
Heliox21	0.8096	0.6169	0.7478	0.1950
Standard	0.8454	0.5460	0.8694	−0.1001

Examining equation (1), the ratio Q_S_/V_E_ provides a measure of the supply gas flow relative to patient demand as discussed above. The ratio between the inspiratory and expiratory times (t_i_/t_e_) was included because with all else held constant, changes to this ratio affect inspiratory flow rates, which play a role in determining the relative resistances of supply and leak flow pathways. The absolute values of the constants *C* and *D*, relative to one another, provide an indication of the relative sensitivity of delivered helium concentrations to these two terms. For all three masks, the ratio Q_S_/V_E_ was the dominant term. Of further interest, the coefficient *D* was opposite in sign for the Heliox21 mask than for the other two masks. That is to say, for the Hi-Ox and standard masks, increasing inspiratory times, associated with lower inspiratory flow rates, tended to increase delivered helium concentrations, whereas for the Heliox21 mask the effect was opposite. This result may be related to the hypothesis provided above, in that for the Heliox21 mask lower flow rates tended to favor flow through leaks over that from the fresh gas supply; however, no firm conclusions should be drawn without a more detailed analysis of the underlying fluid mechanics.

Finally, though the data shown in Figure 8 collapse tightly when plotted against the two ratios, Q_s_/V_E_ and t_i_/t_e_, it is important to note that our methodology included a procedure to fit the masks to the face model in a reproducible manner. Clearly, variation in the quality and tightness of fit between the mask cuff and the face will affect delivered gas concentrations, and this variation is not captured in the experiments reported here.

### Suggestions for improved face models

While the bench data reported herein were in reasonable agreement with available clinical data, a number of points could be improved in future test designs. First, even though masks were adjusted to produce as good a fit as possible, the delivered helium concentrations determined using the Laerdal and Simulaids models tended to fall near, or in some cases even below, the lower range of values measured in humans. Some discrepancy may be expected due to variation in individual subjects’ breathing patterns around the average values that were reproduced on the bench; however, tactile examination of the face models suggests that they are both smoother and harder than a typical human face. Given that neither face model was specifically designed for the purpose of mask testing, it may be that a purpose-built model, for example one that simulates a layer of soft tissue over a hard bone-like structure, will allow clinical mask performance to be more accurately reproduced. Borrowing from evaluation of masks used for aerosol delivery, such a model could also allow delivered gas concentrations to be assessed as a function of the force with which the mask is applied to the face [19,20,22], with an ideal mask maintaining high concentrations at low force. To enable such assessment, a purpose-built model should include the entire head (in contrast to the Simulaids face model selected for the present study) given that facemasks used for gas delivery include a variety of straps or harnesses that secure around the back of the head. Potential differences between oral and nasal breathing also merit investigation, especially for applications where variation in breathing route over time or between patients is expected.

## Conclusions

As new applications of medical gases reach the stage of clinical evaluation, the need for novel patient interfaces and gas supply means will increase. In the present work, two face manikins normally used in life support training were incorporated into a bench top model for assessing delivery of He/O_2_ through facemasks, and shown to reproduce available clinical data with reasonable accuracy. Experiments were then conducted to explore variation in delivered helium concentrations between three non-rebreather facemasks, and large differences in mask performance were identified. With continued refinement, it is anticipated that the availability of reliable benchtop methods for evaluating facemask and other patient interface designs will help to accelerate the development process and assist in demonstrating clinical effectiveness of medical gases.

### Appendix

The error function is a mathematical function of the form

$$\mathrm{erf}\left(x\right)=\frac{2}{\sqrt{\pi }}\int_{0}^{x}e^{-t^{2}}\mathrm{dt}$$

which owes its name to its original use in measurement theory, but is now used more widely in various branches of mathematics and engineering.

The error function is included as a built-in function in most mathematical software packages, and is available in Microsoft EXCEL after installation of the Analysis ToolPak add-in.

## Competing interests

At the time of the study, all authors were employed by Air Liquide, a company that produces helium/oxygen mixtures and is conducting clinical trials to assess their efficacy in the management and treatment of respiratory diseases.

## Authors’ contributions

AM conceived of the study, designed the experiments, participated in performing the bench experiments, and wrote the manuscript. IK participated in the conception and design of the study, and edited the manuscript. YL participated in the design of experiments, performed the majority of bench experiments, and contributed to drafting the manuscript. KT participated in the design of experiments, and created the data acquisition system. GC participated in the design of the experiments. JT participated in the conception and design of the study, and edited the manuscript. All authors read and approved the final manuscript.
